# Humoral Response of Patients With Autoimmune Rheumatic Disease to BNT162b2 Vaccine: A Retrospective Comparative Study

**DOI:** 10.7759/cureus.24585

**Published:** 2022-04-29

**Authors:** Omar Alsaed, Samar AL Emadi, Eman Satti, Bassam Muthanna, Safna Farsana Akkam Veettil, Hadeel Ashour, Prem Chandra, Einas A. Alkuwari, Peter Coyle

**Affiliations:** 1 Medicine Department, Rheumatology Division, Hamad Medical Corporation, Doha, QAT; 2 Medical Research Center, Hamad Medical Corporation, Doha, QAT; 3 Department of Laboratory Medicine and Pathology, Hamad Medical Corporation, Doha, QAT

**Keywords:** rituximab, humoral immune response, disease modifying antirheumatic drug, autoimmune rheumatic disease, anti-sars-cov-2 vaccine

## Abstract

Objective

The effectiveness and safety of SARS-CoV-2 vaccines in patients with autoimmune rheumatic diseases (ARDs) treated with immunomodulators remain uncertain. Therefore, this study aimed to evaluate whether the humoral immune response to the BNT162b2 vaccine differs between patients without and with ARDs treated with immunomodulators.

Methods

We retrospectively reviewed 3208 electronic medical records from the database of the Hamad Medical Corporation (HMC) outpatient rheumatology clinics to capture patients with ARDs and control patients without autoimmune inflammatory diseases. All patients who were SARS-CoV-2 infection-naïve, had received two doses of BNT162b2 vaccination, and had been serologically tested using Elecsys® anti-SARS-CoV-2 S immunoassays (Roche Holdings AG, Basel, Switzerland), were included in the analysis. Patients with ARD were classified into six subgroups according to the received ARD immunomodulators: methotrexate monotherapy (MTXM), a combination of conventional synthetic disease-modifying antirheumatic drugs (Cs-DMARDs), tumor necrosis factor inhibitor (TNF-i), rituximab, interleukin-6 inhibitor (IL6-i), and Janus kinase inhibitor (JAK-i). Samples with an anti-SARS-CoV-2 S titer of <0.8 and <132 binding antibody unit (BAU)/mL were defined as negative and poor seroconversion, respectively. The overall mean of anti-SARS-CoV-2 S titer and its level at <0.8 and <132 were compared between the six subgroups of patients with ARD and the controls by performing an unpaired *t*-test and Chi-squared or Fisher's exact test as appropriate.

Results

The mean (SD) age of 110 patients with ARDs and 20 controls was 47.1 (12) and 59.3 (8.9) years (P < 0.001), respectively, and women predominated both groups (60% vs*.* 75%, P = 0.20). The most frequently prescribed Cs-DMARDs was methotrexate in 50 (45.5%) patients, followed by TNF-i in 46 (41.8%), rituximab in 20 (18.2%), JAK-i in 12 (10.9%), and IL6-i in 7 (6.4%) patients. The mean (SD) anti-SARS-CoV-2 S antibody titer of only the rituximab subgroup significantly differed from the controls (P = 0.012).

Conclusion

The most prevalent ARD immunomodulators (Cs-DMARDs, TNF-i, JAK-i, and IL6-i) were associated with comparable seroconversion rates to the BNT162b2 vaccine. In comparison, rituximab was significantly associated with decreased immunogenicity.

## Introduction

Owing to the availability of different SARS-CoV-2 vaccines, the responses of patients with autoimmune rheumatic diseases (ARDs) to the emerging vaccines remain uncertain. The BNT162b2 vaccine (Pfizer Inc., NY, USA; BioNTech SE., Mainz, Germany), which was the first mRNA-based vaccine approved by the FDA for treating SARS-CoV-2, initially appeared effective and safe. However, it is unclear whether the same applies to patients with ARDs, as early studies on BNT162b2 did not include patients with ARDs and immunocompromised patients [1,2]. Nevertheless, data in this regard have been gradually accumulating. The immunogenicity of solid organ transplant recipients was reportedly reduced after immunization with mRNA-based vaccines, particularly among patients treated with glucocorticoids and antimetabolite medications, such as mycophenolate [3-6]. Advances in immune therapies have paved the way for the use of immunomodulators and anti-cytokine agents such as CD20-depleting agents and inhibitors of Janus kinase (JAK-i), tumor necrosis factor (TNF-i), and interleukin-6 inhibitors (IL6-i) to treat ARDs. These agents are considered immunomodulators rather than immunosuppressors. Our understanding of the immunogenicity of SARS-CoV-2 mRNA-based vaccines in patients with ARDs receiving such agents has been improving.

Theoretical analyses and our experience with influenza and pneumococcus vaccines indicate that serological responses to SARS-CoV-2 vaccines are low in immunocompromised patients [7-11]. International rheumatology societies have released recommendations for multidisciplinary panels of rheumatologists, infectious disease specialists, and public health experts regarding new emerging SARS-CoV-2 vaccines for patients with ARDs and immunocompromised patients. These recommendations are intended to provide direction to treatment providers for patients with rheumatic diseases on how to best apply SARS-CoV-2 vaccines. The recommendations by these panels are based on the use and timing of immunomodulatory medications according to evidence extrapolated from their immunologic effects as the medications are related to other vaccine types [12,13]. These recommendations are continuously updated with the availability of new evidence. Reports on this topic are scant, but it is essential to accumulate data from various populations in different parts of the world for formulating global guidance and recommendations.

In Qatar, a COVID-19 vaccination campaign was started in December 2020, using the first SARS-CoV-2 vaccine, BNT162b2, an mRNA-based vaccine. In this retrospective study, we compared humoral responses of patients with ARDs treated with immunomodulators and patients without autoimmune inflammatory diseases to BNT162b2.

## Materials and methods

Study population

Patients who had ARDs but were SARS-CoV-2 infection-naïve, had two doses of BNT162b2 vaccine, and had been tested for anti-SARS-CoV-2 S IgG antibodies at least two weeks after the second vaccination were identified. Control patients included were SARS-CoV-2 infection-naïve and met the same criteria as the patients with ARDs but without ARDs. The exclusion criteria for both study groups included patients who were previously confirmed for COVID-19 infection by positive polymerase chain reaction (PCR) results of nasopharyngeal swabs, had positive serum nucleocapsid protein antibodies of SARS-CoV-2, had coexisting malignancies, HIV infection, and were on immunosuppressive therapy for other indications, such as organ transplantation. In June 2021, 3208 electronic medical records from the electronic database of the Hamad Medical Corporation (HMC) outpatient rheumatology clinics were used to identify both study groups according to the defined inclusion and exclusion criteria. These records of these patients were then retrospectively reviewed to capture demographic data, including age, sex, ethnicity, BMI, ARD medications, date of administration of the two doses of BNT162b2, and comorbidities, including diabetes mellitus, hypertension, chronic kidney disease, chronic heart disease, and underlying ARDs.

The patients with ARDs were assigned to six subgroups according to their ARD medications: methotrexate monotherapy (MTXM), a combination of conventional synthetic disease-modifying antirheumatic drugs (Cs-DMARDs), TNF-i, rituximab, IL6-i, and JAK-i.

Anti-SARS-CoV-2 humoral response tests

Anti-SARS-CoV-2 humoral responses were assessed using Elecsys® kits (Roche Holdings AG, Basel, Switzerland), which measure titers of anti-SARS-CoV-2 S IgG antibodies. The results were defined as negative seroconversion and poor seroconversion when the titers were <0.8 and <132 binding antibody unit (BAU)/mL, respectively, which is the initial cut-off titer used to qualify plasma donors who convalesce after SARS-CoV-2 infection.

Ethical approval

The ethical review board of HMC approved this study (protocol number 01-21-584), and the study was performed in accordance with the relevant guidelines and regulations. The need for informed consent from the patients was waived as relevant data were extracted from patients' medical records and rendered innominate. The study did not involve patient interviews or extra blood sampling.

Statistical analysis

Categorical and continuous values are expressed as frequency (%) and mean ± SD or as median with interquartile range, as appropriate. Descriptive statistics are used to summarize demographics, immunomodulators and immunosuppression agents, underlying rheumatic disease, antibody titer (humoral response), and other clinical data such as comorbidities (diabetes, hypertension, chronic kidney, and cardiovascular diseases). Associations between two or more qualitative variables (demographic, clinical, and other related characteristics of the participants) were examined using Chi-squared or Fisher's exact test as appropriate. The mean of anti-SARS-CoV-2 S titer, negative seroconversion (titer <0.8 BAU/mL), and poor seroconversion (titer <132 BAU/mL) were compared between the six subgroups of patients with ARD and the controls. This was done by performing an unpaired t-test or Mann-Whitney U test and Chi-squared or Fisher's exact test, as appropriate. Factors associated with negative seroconversion and poor seroconversion were determined by performing a binary logistic regression analysis. All p-values are two-tailed, and results with p < 0.05 were considered significant. All data were statistically analyzed using SPSS 27 (IBM Corp., Armonk, NY, USA).

## Results

Baseline characteristics of the study population

We analyzed the data of 110 patients with ARDs and 20 controls. Sex distribution was balanced between the groups, and females were predominant (60% vs. 75%, P = 0.20). The patients in the control group were older than those in the ARD group (mean age [SD], 59.3 [8.9] vs. 47.1 [12] years; P < 0.0001). The incidence rate of hypertension, dyslipidemia, chronic kidney disease, chronic heart disease, hypothyroidism, and obesity did not significantly differ between the groups. In contrast, diabetes mellitus was more prevalent in the controls than in patients with ARDs (15 [13.6%] vs. 8 [40%], P = 0.004).

Among patients with ARDs, the most prevalent ARD was rheumatoid arthritis (54%), followed by ankylosing spondylitis (20.9%), psoriatic arthritis (9.1%), systemic lupus erythematosus (6.3%), vasculitis (2.7%), inflammatory myositis (1.8%), Sjögren disease (1.8%), systemic sclerosis (0.9%), IgG4-related disease (0.9%), and adult-onset Still disease (0.9%). Furthermore, 50 (45.5%), 26 (23.6%), 46 (41.8%), 20 (18.2%), 12 (10.9%), and 7 (6.4%) patients with ARDs were on MTXM, Cs-DMARDs, TNF-i, rituximab, JAK-i, and IL6-i, respectively. The mean interval (SD) between the last rituximab dose and second vaccination for patients with ARDs was 7.6 (4.5) months. Table 1 shows the baseline demographic and clinical characteristics of the study cohort, ARD diagnosis, and ARD medications used.

**Table 1:** Baseline demographic and clinical characteristics of study groups. ARD: Autoimmune rheumatic disease; Cs-DMARDs: Conventional synthetic disease-modifying antirheumatic drugs; IL6-i: Interleukin 6 inhibitor; JAK-i: Janus kinase inhibitor; TNF-i: Tumor necrosis factor inhibitor. *Some patients were with more than one comorbidity and were prescribed more than one medication.

	Controls (N = 20)	ARD (N = 110)	P-value
Mean age (SD) years	59.3 (8.9)	47.1 (12)	0.000
Sex N (%)			0.203
Female	15 (75)	66 (60)	
Male	5 (25)	44 (40)	
Ethnicity (p)			0.151
Arab N (%)	17 (85)	72 (80.9)	
Asian N (%)	1 (5)	26 (23.6)	
Other N (%)	2 (10)	12 (10.9)	
Comorbidities*, N (%)			
Diabetes mellitus	8 (40)	15 (13.6)	0.004
Hypertension	7 (35)	27 (24.5)	0.328
Dyslipidemia	5 (25)	13 (11.8)	0.116
Chronic kidney disease	1 (5)	2 (1.8)	0.397
Chronic heart disease	1 (5)	5 (4.6)	0.936
Hypothyroidism	1 (5)	10 (9.1)	0.545
Obesity	3 (15)	14 (12.7)	0.782
None	9 (45)	59 (53.6)	0.477
ARD diagnosis N (%)	NA		NA
Rheumatoid arthritis		60 (54)	
Psoriatic arthritis		10 (9.1)	
Ankylosing spondylitis		23 (20.9)	
Systemic lupus erythematosus		7 (6.3)	
Sjögren disease		2 (1.8)	
Inflammatory myositis		2 (1.8)	
Systemic sclerosis		1 (0.9)	
Vasculitis		3 (2.7)	
Other		2 (1.8)	
ARD medications* N (%)	NA		NA
Methotrexate		50 (45.5)	
Hydroxychloroquine		20 (18.2)	
Leflunomide		4 (3.6)	
Sulfasalazine		6 (5.5)	
Cs-DMARDs		26 (23.6)	
TNF-i		46 (41.8)	
JAK-i		12 (10.9)	
Rituximab		20 (18.2)	
Mean time (SD) between rituximab dose and second vaccine dose, months		7.6 (4.5)	
IL6-i		7 (6.4)	
Mycophenolate		3 (2.7)	
Glucocorticoids (mg/day)		9 (8.2)	
≥ 10		4 (3.6)	
< 10		5 (4.5)	

Humoral responses to BNT162b2 vaccine

Patients with ARDs treated with Cs-DMARDs, MTXM, TNF-i, JAK-i, and IL6-i, showed immune responses comparable to those of the controls. The mean (SD) anti-SARS-CoV-2 S IgG titers of the patients with ARDs were 238 (34.3) for Cs-DMARDs, 230 (44) for MTXM, 211 (80) for TNF-i, 208 (76.2) for JAK-i, and 217 (86.8) for IL6-i compared with 226 (57.5) for control (P = 0.39, 0.84, 0.44, 0.45, and 0.74, respectively). Only patients with ARDs treated with rituximab had a lower humoral response than the control group (P = 0.012, 95% CI: 18.731-139.940). Rituximab was associated with a lower seropositivity rate than the control (65% vs. 100%), and higher incidence of poor seroconversion (<132 BAU/mL) compared to the control (40% vs. 10%). The SARS-CoV-2 S antibody was undetectable in seven patients with ARDs, and these patients were treated with rituximab. Table 2 shows the immune responses of patients with ARDs treated with immunomodulators and controls with the BNT162b2 vaccine.

**Table 2:** Comparison of immune responses to BNT162b2 vaccine between patients with ARDs treated with immunomodulators and the controls. IL6-i: Interleukin 6 inhibitor; JAK-i: Janus kinase inhibitor; MTXM: Methotrexate monotherapy; Cs-DMARDS: Combinations of conventional synthetic disease-modifying antirheumatic drugs; TNF-i: Tumor necrosis factor inhibitor; sc: Seroconversion; NA: Not applicable. Anti-SARS-CoV-2 S titers: ^∫^ ≥ 0.8 BAU/mL, *<132 BAU/mL and ^†^< 0.8 BAU/mL.

	Control (n=20)	Cs-DMARDs (n=26)	MTXM (n=15)	TNF-i (n=46)	Rituximab (n=20)	JAK-i (n=12)	IL6-i (n=7)
Mean, SARS-CoV-2 S IgG (p-value)	226.8 (Ref)	238.7 (0.389)	230.5 (0.841)	211.5 (0.443)	147.5 (0.012)	208.7 (0.449)	217.1 (0.740)
^∫^Seropositive, (%) p-value	(100) Ref	(100) NA	(100) NA	(100) NA	(65) 0.004	(100) NA	(100) NA
*Poor sc (%) p-value	(10) Ref	(3.8) 0.572	(6.7) 1.000	(19.6) 0.338	(40) 0.028	(25) 0.338	(14.3) 1.000
^†^ Negative sc (%) p-value	(0) Ref	(0) NA	(0) NA	(0) NA	(35) 0.004	(0) NA	(0) NA

Medications to treat ARDs associated with poor and negative seroconversion reactions to the BNT162b2 vaccine

Seven patients with ARDs had an anti-SARS-CoV-2 S IgG titer of zero. Rituximab, mycophenolate, and glucocorticoid users were significantly associated with negative seroconversion from the BNT162b2 vaccine (P ≤ 0.0001, 0.001, and <0.0001, respectively). However, only three patients were treated with mycophenolate, and therefore, a solid conclusion cannot be made about this drug. Immunogenicity to the BNT162b2 vaccine was poor in 21 patients with ARDs, as the anti-SARS-CoV-2 S IgG titer was <132 BAU/mL. Rituximab and glucocorticoid were significantly associated with a poor response to the BNT162b2 vaccine (P = 0.009 and <0.0001, respectively) (Table 3).

**Table 3:** Medications to treat ARD associated with poor and negative seroconversion reactions to the BNT162b2 vaccine. Ab: Antibody; ARD: Autoimmune rheumatic disease; Cs-DMARDs: Conventional synthetic disease-modifying antirheumatic drugs; IL6-i: Interleukin 6 inhibitor; JAK-i: Janus kinase inhibitor; MTX: Methotrexate; MTXM: Methotrexate monotherapy; TNF-i: Tumor necrosis factor inhibitor; NA: Not applicable.

Medications	Seropositive Ab titre ≥ 0.8 (n = 103)	Negative seroconversion Ab titre < 0.8 (n = 7)	P-value	Seropositive Ab titre ≥ 132 (n = 89)	Poor seroconversion Ab titre < 132 (n = 21)	P-value
MTXM	15 (14.6)	0 (0)	0.590	14 (15.7)	1 (4.8)	0.294
MTX combination	48 (46.6)	2 (28.6)	0.452	40 (44.9)	10 (47.6)	0.825
Hydroxychloroquine	18 (17.5)	2 (28.6)	0.609	18 (20.2)	2 (9.5)	0.353
Sulfasalazine	6 (5.8)	0 (0)	1.000	6 (6.7)	0 (0)	0.593
Leflunomide	4 (3.9)	0 (0)	1.000	2 (2.2)	2 (9.5)	0.164
Cs-DMARDs only	26 (25.2)	0 (0)	0.195	25 (28.1)	1 (4.8)	0.023
Mycophenolate	1 (1)	2 (28.6)	0.010	1 (1.1)	2 (9.5)	0.093
TNF-i	46 (44.7)	0 (0)	0.040	37 (41.6)	9 (42.9)	1.000
TNF-i monotherapy	24 (23.3)	0 (0)	0.343	22 (24.7)	2 (95)	0.154
JAK-i	12 (11.7)	0 (0)	1.000	9 (10.1)	3 (14.3)	0.696
Rituximab	13 (12.6)	7 (100)	0.000	12 (13.5)	8 (38.5)	0.009
IL6-i	7 (6.8)	0 (0)	1.000	6 (6.7)	1 (4.8)	1.000
Glucocorticoid	4 (3.9)	5 (714)	0.000	2 (2.2)	7 (33.3)	0.000

Clinical characteristics and factors associated with poor and negative seroconversion responses to the BNT162b2 vaccine

Patients aged <40 years diagnosed with autoimmune connective tissue or chronic kidney diseases were significantly associated with undetectable anti-SARS-CoV-2 S antibodies (P = 0.032, <0.0001, and 004, respectively). Table 4 shows factors associated with poor and negative seroconversion responses to the BNT162b2 vaccine.

**Table 4:** Factors associated with poor and negative seroconversion responses to the BNT162b2 vaccine. ARDs: Autoimmune rheumatic diseases; Ab: Antibody.

Medications	Seropositive Ab titer ≥ 0.8 (n = 103)	Negative seroconversion Ab titer < 0.8 (n = 7)	P-value	Seropositive Ab titer ≥ 132 (n = 89)	Poor seroconversion Ab titer < 132 (n = 21)	P-value
Age (years)						
< 40	30 (29.1)	5 (71.4)	0.032	26 (29.2)	9 (42.9)	0.227
40-49	31 (30.1)	1 (14.3)	0.671	29 (32.6)	3 (14.3)	0.115
≥ 50	42 (40.8)	1 (14.3)	0.243	34 (38.2)	9 (42.9)	0.805
Sex N (%)						
Female	60 (58.3)	6 (85.7)	0.239	54 (60.7)	12 (57.1)	0.766
Male	43 (41.7)	1 (14.3)		35 (39.3)	9 (42.9)	
Comorbidities N (%)						
Diabetes mellitus	13 (12.6)	2 (28.6)	0.243	11 (12.4)	4 (19)	0.480
Hypertension	24 (23.3)	3 (42.9)	0.359	20 (22.5)	7 (33.3)	0.298
Dyslipidaemia	12 (11.7)	1 (14.3)	1.000	12 (13.5)	1 (4.8)	0.456
Chronic kidney disease	0 (0)	2 (28.6)	0.004	0 (0)	2 (95)	0.004
Chronic heart disease	3 (2.9)	1 (14.3)	0.236	3 (3.4)	1 (4.8)	0.581
Hypothyroidism	9 (8.7)	1 (14.3)	0.497	8 (9)	2 (9.5)	1.000
Obesity	12 (11.7)	2 (28.6)	0.218	12 (13.5)	2 (9.5)	1.000
ARDs N (%)						
Rheumatoid arthritis	30 (29.1)	2 (28.6)	0.154	51 (57.3)	9 (42.9)	0.232
Connective tissue disease	31 (30.1)	5 (71.4)	0.000	10 (11.2)	5 (23.8)	0.158
Spondyloarthropathy	42 (40.8)	0 (0)	0.100	26 (29.2)	7 (33.3)	0.711
Other	30 (29.1)	0 (0)	1.000	2 (2.2)	0 (0)	1.000

All seven patients with negative seroconversion in response to BNT162b2 were administered rituximab; five were administered glucocorticoids; and two each were administered mycophenolate, methotrexate, and hydroxychloroquine. The humoral response was good (titer > 132 BAU/mL) in 12 patients treated with rituximab and poor in one patient (titer < 132 BAU/mL). The mean (SD) interval between the last rituximab dose and the second vaccination for seropositive and seronegative patients on rituximab did not significantly differ (7.9 (4.4) vs. 7.0 (5) months, P = 0.68) (Table 5).

**Table 5:** Clinical characteristics of patients with negative seroconversion after immunization with the BNT162b2 vaccine (undetectable anti-SARS-CoV-2 S titers). ANCA: Antineutrophil cytoplasmic antibodies; CHD: Chronic heart disease; CKD: Chronic kidney disease; d: Day; DM: Diabetes mellitus; ESRD: End-stage renal disease; HTN: Hypertension; ILD: Interstitial lung disease; SLE: Systemic lupus erythematosus; RA: Rheumatoid arthritis; SS: Systemic sclerosis; F: Female; w: Week.

Patient No.	Sex/age (y)	Comorbidities	ARD	ARD medications	Rituximab to vaccine interval (d)
1	F, 55	HTN, DM, obesity, dyslipidaemia	RA	Rituximab 1 g × 2, Methotrexate 10 mg/w	158
2	F, 36	ILD	SS/RA overlap syndrome	Rituximab 1 g × 2, Mycophenolate 1.5 g/d, Prednisolone 5 mg/d	141
3	F, 44	HTN	SLE	Rituximab 1 g × 2, Mycophenolate 2 g/d, Hydroxychloroquine 200 mg/d, Prednisolone 15 mg/d	163
4	F, 31	ESRD, HTN	SLE	Rituximab 250 mg	544
5	F, 39	-	SLE with secondary Sjögren disease	Rituximab 0.5 g × 2, Methotrexate 20 mg/w, Hydroxychloroquine 400 mg/d	199
6	F, 39	HTN, DM, CKD, CHD, dyslipidaemia, hypothyroidism	Polymyositis	Rituximab 1 g × 2, Prednisolone 5 mg/d	141
7	F, 37	Obesity	ANCA associated vasculitis	Rituximab 1 g × 2, Azathioprine 100 mg/d, Prednisolone 5 mg/d	120

## Discussion

Here, the humoral responses to BNT162b2 were comparable between patients without ARDs and with ARDs treated with MTXM, Cs-DMARDs, TNF-i, JAK-i, and IL6-i. In contrast, rituximab was significantly associated with poor seroconversion. These results are consistent with recent findings [14-18]. The anti-SARS-CoV-2 antibody was undetectable in seven (35%) out of 20 patients administered with rituximab, and 65% of these patients were seropositive. Furer V et al. found that 41% of patients treated with rituximab were seropositive [17], whereas 54.4% were seropositive in an Austrian cohort [15]. This difference might result from concomitant medication use and differences in the interval between the last rituximab dose and vaccination, which was comparatively longer in the present study (7.6 vs. 6.9 and 1.7 months). This suggests that increasing the interval between the last rituximab dose and vaccination as much as possible, if the underlying disease is well controlled, or choosing a different treatment option would be beneficial to patients with ARDs treated with rituximab during the current pandemic. In addition, evaluating CD19 levels before administering vaccines might be helpful, as peripheral B-cell depletion is correlated with negative seroconversion [15].

Methotrexate is one of the most popular DMARDs used for treating ARDs. The seropositivity rates after the administration of BNT162b2 were 100% and 96% in our cohort administered MTX alone and MTX in combination with other DMARDs, respectively. Our binary logistic regression analysis did not reveal any association between MTX and poor seroconversion (titer < 132 BAU/mL). These findings contradict the results of two prospective studies in which 62% and 84% of patients treated with MTX were seropositive [17,19]. However, this was consistent with the results of a Dutch study in which seropositivity was comparable between patients and healthy controls after administering an mRNA-based SARS-CoV-2 vaccine [20]. We believe that this discrepancy stems from variations in the timing of anti-SARS-CoV-2 antibody tests. The median interval between the second vaccination and antibody tests was 63 days in the present study and 38 days in a cohort with seropositivity comparable to a control group [20]. Comparing the median interval in studies that reported adequate and inadequate responses revealed that antibody tests within 14 days after vaccination showed inadequate anti-SARS-CoV-2 antibody titers in patients medicated with MTX. This confirmed that MTX might delay rather than prevent seroconversion.

Our study has several strengths. It is among the first few studies to evaluate the effects of the BNT162b2 vaccine in patients with ARDs managed with immunosuppressive agents. Using data from other population cohorts is valuable for generalizing future recommendations. Our cohort comprised patients with various types of ARDs that were usually treated with MTX, TNF-i, and rituximab. Most published studies have been prospective in nature, and the effects of anti-SARS-CoV-2 antibody were tested immediately after vaccination, which might have resulted in inadequate seroconversion. The median interval between completing vaccination and anti-SARS-CoV-2 antibody tests was 60 days in the present study. All our study participants were SARS-CoV-2 infection-naïve, and therefore, our study provided a reasonable estimate of the humoral response to SARS-CoV-2 vaccines in patients with ARD treated with immunosuppressive drugs. However, the following limitations should be addressed. We did not test neutralizing antibodies with anti-SARS-CoV-2 antibody, but the anti-SARS-CoV-2 IgG titers correlated with the levels of neutralizing antibodies [15,21]. Owing to the small number of patients in this study, the results are more exploratory than confirmative. Age, sex, and comorbidities differed between the ARD and control groups. Because of the retrospective nature of this study, data on ARD characteristics such as disease activity and duration of ARDs, which could influence the vaccination response, were lacking. Some patients might have stopped their regular medications around the time of vaccination, which could have affected our results. A prospective study with larger sample size is needed to overcome these drawbacks. Finally, many non-mRNA-based SARS-CoV-2 vaccines have been approved by the FDA and other drug agencies. These vaccines differ in terms of the technology used to induce immunogenicity against SARS-CoV-2. This difference will generally result in different effects and safety in patients with ARDs treated with immunomodulators. However, data on the efficacy of non-mRNA-based SARS-CoV-2 vaccines for ARDs are limited.

## Conclusions

In summary, humoral responses and seroconversion rates after the BNT162b2 vaccination were similar between patients with ARDs treated using Cs-DMARDs, TNF-i, IL6-i, and JAK-i and the controls. Therefore, until an alternative therapy to rituximab is unavailable, SARS-CoV-2 vaccination should be delayed for as long as possible for patients on rituximab if the underlying ARD is controlled. Most available data on the effects of SARS-CoV-2 vaccinations in patients with ARDs describe only the short-term humoral response. However, the sustainability of seroconversion and the need for a booster vaccination in patients using Cs-DMARDs, anti-cytokine agents, and JAK-i, who present good early immunogenicity, should be further investigated in longitudinal studies.
